# MetaAnalyst: a user-friendly tool for metagenomic biomarker detection and phenotype classification

**DOI:** 10.1186/s12874-022-01812-5

**Published:** 2022-12-28

**Authors:** Mustafa Alshawaqfeh, Salahelden Rababah, Abdullah Hayajneh, Ammar Gharaibeh, Erchin Serpedin

**Affiliations:** 1 School of Electrical Engineering and Information Technology, German Jordanian University, Amman, Jordan; 2 Department of Systems Science and Industrial Engineering, State University of New York at Binghamton, Binghamton, NY, USA; 3 Electrical and Computer Engineering Department, Texas A &M University, College Station, TX, USA

**Keywords:** Metagenomics, Biomarker detection, Phenotype classification, Graphical user interface, Software

## Abstract

**Background:**

Many metagenomic studies have linked the imbalance in microbial abundance profiles to a wide range of diseases. These studies suggest utilizing the microbial abundance profiles as potential markers for metagenomic-associated conditions. Due to the inevitable importance of biomarkers in understanding the disease progression and the development of possible therapies, various computational tools have been proposed for metagenomic biomarker detection. However, most existing tools require prior scripting knowledge and lack user friendly interfaces, causing considerable time and effort to install, configure, and run these tools. Besides, there is no available all-in-one solution for running and comparing various metagenomic biomarker detection simultaneously. In addition, most of these tools just present the suggested biomarkers without any statistical evaluation for their quality.

**Results:**

To overcome these limitations, this work presents MetaAnalyst, a software package with a simple graphical user interface (GUI) that (i) automates the installation and configuration of 28 state-of-the-art tools, (ii) supports flexible study design to enable studying the dataset under different scenarios smoothly, iii) runs and evaluates several algorithms simultaneously iv) supports different input formats and provides the user with several preprocessing capabilities, v) provides a variety of metrics to evaluate the quality of the suggested markers, and vi) presents the outcomes in the form of publication quality plots with various formatting capabilities as well as Excel sheets.

**Conclusions:**

The utility of this tool has been verified through studying a metagenomic dataset under four scenarios. The executable file for MetaAnalyst along with its user manual are made available at https://github.com/mshawaqfeh/MetaAnalyst.

**Supplementary Information:**

The online version contains supplementary material available at 10.1186/s12874-022-01812-5.

## Background

Recent advances in high throughput sequencing technologies have opened the door to a new era for genetic studies, called *metagenomics*. In contrast to the conventional cultivation-based approaches, metagenomics enables the characterization of compositional and functional profiles of microbial colonies directly from environmental samples. Increasing number of metagenomic studies have revealed strong associations between the imbalance in microbial abundance profiles and a wide range of diseases such as obesity [1, 2], diabetes [3], inflammatory bowel disease (IBD) [4], and cancer [5, 6]. These results suggest utilizing metagenomic data for identifying potential biomarkers and developing phenotype classification models for microbial-associated diseases.

In addition to the contribution of the biomarkers in understanding the biological process under study, biomarker detection and phenotype predictive models play a central role in translating the embedded information in metagenomic datasets into clinical applications. One potential application is to utilize the detected markers for the development of potential therapies and treatments for microbial related diseases. Another application is to integrate the abundance levels of the suggested biomarkers into a single numeric value, called the *dysbiosis index*, that measures and tracks the disease activity [7, 8].

Therefore, several algorithms and computational tools have been proposed for biomarker detection such as LEfSe [9], RPCA [10], RegLRSD [11], IMG/M [12], MeAtML [13], Fizzy [14], Boruta [15], ENNB [16], MetagenomeSeq [17], MicrobiomeDDA [18], ShotgunFunctionalizeR [19], MetaStats [20], Raida [21], FANTOM [22]. Due to the similarity between metagenomic data sequence-based transcriptomics, tools that were developed originally for analyzing RNA sequencing (RNA-seq) data such as edgeR [23] and DESeq2 [24] can be applied to analyzing metagenomic data. In addition, conventional standard hypothesis testing (e.g., chi-squared, log-t, t-test, Leven Quadrati, Leven absolute, Wilcoxon rank sum, Brown-Forythe, Welch and Kolmogorov-Smirnov) and feature selection techniques (e.g., ReliefF [25], Pearson correlation [26], BSS/WSS [26]) have been suggested for finding differentially abundant microbes in metagenomic data. It is worth to mention that there exist various general purpose (i.e., not dedicated to metagenomic) tools that packed several feature selection techniques to find the important features (markers) such as FeatureSelect [27] and MetaFS [28]. Table 1 summarizes the characteristics of these tools.

However, the majority of these tools lack a user-friendly interface, and more challenging, most of them are of command-line nature, which is less comfortable compared to graphical user interface (GUI)-based software. Besides, these methods were developed using different programming languages (R, Python, C, C++, Matlab, etc), and their operation requires handling version compatibility and package dependencies related issues. Therefore, installing, configuring, and running these tools present sometimes a serious challenge for researchers with limited background in such professional soft skills. This problem becomes more challenging in scenarios where these tools are required to be installed on a large number of workstations (e.g., educational and research laboratories) [29]. One further limitation of several existing tools is that they accept one or few file types (e.g., xlsx, txt, biom). Even more, some tools require the data to be arranged in a certain format in the input data file. This decreases the comfort of utilizing these tools, especially when dealing with human-unreadable files such as biom files.

**Table 1:** The characteristics of existing packages for biomarker detection. Y: Yes, N: No, CL: Command Line

Tool	Language	Interface	Input Files	Flexible Data Format	Flexible Study Design	Preprocessing	Evaluate Biomarkers	Run Multiple Algorithms
Boruta [15]	-R	-CL	-R frame	N	N	N	N	Y
edgeR [23]	-R	-CL	-R frame	N	N	-Filtering	Y	N
						-Normalization		
DESeq2 [24]	-R	-CL	-R frame	N	N	N	N	N
ENNB [16]	-R	-CL	-R frame	N	N	-Normalization	N	N
MetagenomeSeq [17]	-R	-CL	- R frame	N	N	-Normalization	N	N
MicrobiomeDDA [18]	-R	-CL	-R frame	N	N	-Normalization	N	N
Shotgun- FunctionalizeR [19]	-R	-CL	- R frame	N	N	N	N	Y
MetaStats [20]	-R	-CL	-R frame	N	N	-Normalization	N	N
Raida [21]	-R	-CL	-R frame	N	N	N	N	N
LEfSe [9]	-Python	-CL -Web	-Tabular	Y	Y	N	N	N
MetaML [13]	-Python	-CL	-tsv	N	N	N	Y	N
Fizzy [14]	-Python	-CL	-biom -csv	N	N	N	N	N
“stats” package of R [30]	-R	-CL	-R frame	N	N	N	N	N
FANTOM$^*$ [22]	-Python	-GUI	-txt	NA	N	N	N	N
STAMP [31]	-Python	-GUI	-tsv	N	N	-Filtering	N	N
XIPE-TOTEC [32]	-Python	-CL -Web	-tsv	N	N	N	N	N
Microbiome Analyst [33]	-Java Script	-Web	-txt -csv -biom	N	N	-Filtering -Scaling -Normalization -Transformation	Y	N
	-Java							
	-R							
METAREP [27]	-Python -Java -Matlab	-CL -GUI	-txt -xlsx -mat	N	N	N	Y	N
DAME [34]	-R	-Web	-biom -csv -trf -HDF5 -JSON	N	Y	-Filtering	Y	N
ShinyMB [35]	-R	-Web	-csv -tsv	N	N	-Stratification	Y	N
MetaFS [28]	-Web server	-Web	-csv	N	N	-Centering -Transformation -Scaling -Normalization	Y	N
RPCA$^*$ [10]	-Matlab -C	-CL	-xls	N	N	N	N	N
RegLRSD [11]	-Matlab -C	-GUI	-xls	N	N	N	N	N
MetaAnalyst	-Matlab	-GUI	-csv -tsv -xls -mat -biom -json	Y	Y	-Scaling -Normalization -Centering	Y	Y

$^{*}$ The implementation of the code is not available

Another challenge is the lack of an *all-in-one* solution that provides a researcher in the field of metagenomics with an easy solution to conduct analysis over multiple tools simultaneously. This feature becomes more demanding if it is combined with the fact that there is no golden method that provides reliable results over all datasets. Specifically, the lists of potential markers that are generated by different algorithms vary significantly [10, 11, 36]. This variation stems from the underlying assumptions behind each method and the characteristics of the input data. Thus, using a variety of biomarker detection algorithms is crucial to enable the researcher to explore the biological problem from different angles (i.e., different algorithms may suggest different markers). Therefore, it is informative to obtain and compare the suggested signatures of several meteganomic biomarker detection techniques simultaneously. To achieve this goal, the current standard approach is to install and run the targeted tools individually. Then, combine the obtained results from these tools manually to generate comparison tables and figures. This involves an additional burden and consumes a significant amount of time and effort.

Besides, the majority of existing tool for metagenomic biomarker discovery lack the flexibility of reformatting the input dataset to enable studying the data under different conditions. In particular, the fundamental step for comparative analysis such as biomarker detection and phenotype classification is to divide the samples into positive and negative classes based on their class labels. Typically, biological samples are annotated with several kinds of information such as health status, body site location, gender. In order to construct the positive and negative cohorts, existing tools with one-level labeling capability enable the user to conduct comparative analysis with respect only to one criterion. To illustrate, assume that the health status (i.e., healthy or diseased) and gender information of the samples are available. With one-level labeling capability, a researcher can directly compare healthy and diseased samples (irrespective of the gender) or to compare male and female subjects (irrespective of the health status). In other words, the user can divide the samples into positive and negative groups based only on one criterion (either health status or gender). However, if the user is interested in creating positive and negative cohorts by combining the two criteria (i.e., health status and gender), then the researcher needs to do this manually. For example, assume that it is required to compare healthy and diseased females (male samples are excluded), then the researcher needs to manually divide the samples into two groups. The first group represents the positive class and it is composed of “diseased and female” subjects, while the second group compromises the “healthy and female” samples and represents the negative class. This one-level labeling becomes more inconvenient to provide flexible study design if the original data includes several levels of labels and the researcher is interested in studying various scenarios (by combining different levels of samples’ labels).

**Fig. 1:**
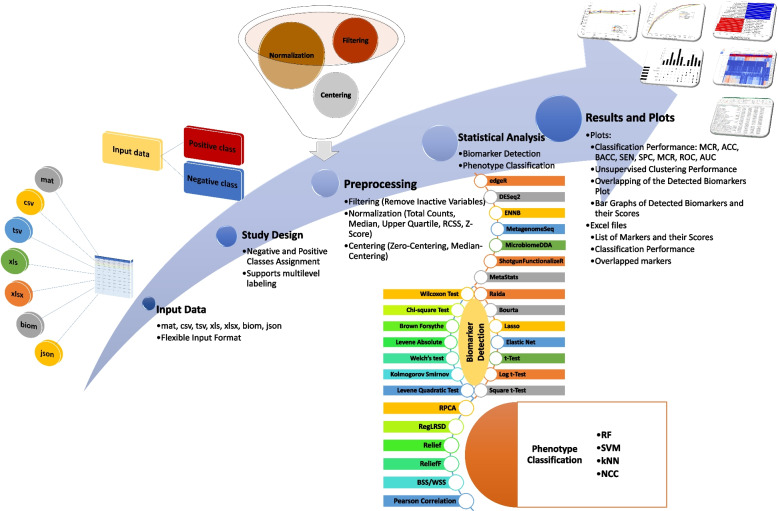
The general workflow of MetaAnalyst is composed of five steps. Step 1: input data. Step 2: study design. Step 3: preprocessing. Step 4: statistical analysis. Step 5: results and plots

One additional serious limitation of several existing tools is that they provide the researcher with the suggested list of biomarkers without performance assessment. In the field of metaproteomics, researchers have payed special attention for evaluating the suggested markers. For example, the authors in [28] have designed an online tool, named MetaFS, that is designed to evaluate the performance of 13 metaproteomics biomarker detection algorithms using four evaluation criteria (clustering, classification, consistency, and prediction of spiked protein). As an additional example, the authors in [37] have conducted comprehensive assessment to 14 biomarker detection algorithms using two criteria: (i) classification power, and (ii) spiked protein discovery. Therefore, it is crucial to develop a user friendly tool to quantify the quality of the detected metagenomic biomarkers.

In order to provide a remedy for these challenges and to improve the efficiency of metagenomic analysis, this work proposes *MetaAnalyst*, an all-in-one standalone package equipped with a user friendly graphical user interface. MetaAnalyst automatically installs and configures 28 tools designed specifically for metagenomic biomarker detection. In addition, MetaAnalyst package includes 4 classifiers, namely support vector machines (SVM), random forest (RF), nearest centroid (NC), and k-nearest neighbor (kNN). These classification methods enable the researcher to go beyond the basic biomarker detection functionality to build complete phenotype classification models and to evaluate the discrimination power of the detected markers. MetaAnalyst provides a variety of metrics to asses the different aspects of classification performance. As a single criterion is not efficient to capture the overall performance of BD algorithms [38], in addition to the classification power, MetaAnalyst captures the unsupervised clustering performance [39] of the detected markers (visualized as two-way dendrogram plots) and the overlapping of the detected biomarkers across multiple BD algorithms (visualized as upset plot).

Furthermore, MetaAnalyst runs and evaluates, simultaneously, any subset of the 28 packed biomarker detection tools and compare their results directly. In contrast to the one-level labeling strategy, MetaAnalyst supports the *multilevel labeling* feature, through which researchers are able to define the positive and negative classes as any logical combination of up to three levels of labels to enable flexible study design. From input perspective, MetaAnalyst accepts 7 different types of input files. In addition to the publication-quality figures, the obtained results are reported as Excel tables to provide the user with further flexibility to generate other kinds of figures.

It is worth to mention that there are some other computational tools, platforms and projects available in the field of analyzing metagenomic data, such as Bioconda [40], Megan [41], UniFrac [42], CAMERA [43] and Galaxy [44]. However, each introduced work tackles an aspect different than the contribution of this paper. For instance, Bioconda [40] is a repository of bioinformatics packages and CAMERA [43] is a community database project that aims to collect metagenomic data and bioinformatics tools to make them widely available to the research community.

## Implementation

The workflow of MetaAnalyst can be divided into five main steps as shown in Fig. 1. To facilitate the analysis, each main step is represented by one tab of the MetaAnalyst software. These tabs are designed to self-guide the user smoothly through the analysis. The following subsections describe these tabs (i.e., steps). Further details with a step-by-step example are available in the software manual https://github.com/mshawaqfeh/MetaAnalyst.

### Step 1: Input data

In general, metagenomic data files are composed of two parts: (1) numerical data, and (2) metadata. The numerical data represents the abundance levels of the operational taxonomic units (OTUs) across all samples. In metagenomics assays, each OTU represents a cluster of similar variants of the 16S rDNA marker gene sequence. Hence, each cluster (i.e., OTU) represents one bacterial species or genus. The second part, which is metadata, contains descriptive information about data such as OTU names, sample IDs, sample labels (e.g., disease/health status, body site location, ethnicity, gender).

The main tasks of this step are (1) to upload the input data file and (2) to extract the numerical data and their associated metadata. Regarding uploading the data, the user needs only to browse existing files on her/his local machine to locate the input file. In order to provide users with higher flexibility, the MetaAnalyst package is designed to support seven different types of input files: mat (Matlab file), csv, tsv, xls, xlsx, biom (Biological Observation Matrix) and json (JavaScript Object Notation). This feature is important to support the all-in-one feature of the MetaAnalyst software by reducing the dependency on other utilities/tools to handle specific input formats such as biom and json files. Upon loading the file, the MetaAnalyst automatically converts it into tabular format to facilitate extracting the abundance levels data and the samples’ labels.

To extract these information, the user needs only to specify their location (rows and columns) in the input file. To simplify this task, the MetaAnalyst package automatically displays the content of the input file directly after selecting the input file in a table within the main window of the MetaAnalyst package. Therefore, users can directly specify the required information to extract different parts of the data without the need to open the original input files externally (using other tools). This feature is especially useful when dealing with “biom” files since such files are not human readable, and typically they require special tools to convert them into readable format. Again, this embedded display of the input data enhances the all-in-one experience. Unlike most existing packages that assume input files to follow specific templates (e.g., the data is column-wise and the variable names are listed in the first column), the MetaAnalyst package is flexible to handle different styles for the input files.

### Step 2: Study design

The first step in comparative-based analysis, such as biomarker detection and phenotype classification, is to construct the positive and negative cohorts. The majority of existing tools perform this division based only on one criterion, commonly the health status (i.e., negative class represents healthy subjects while positive class represents diseased samples). On the other hand, the MetaAnalyst package supports a multilevel labeling strategy that enables researchers to combine several criteria for classifying the samples into positive and negative groups. In particular, a researcher is able to define the positive and negative classes as any logical combination of up to three levels of labels. This flexibility in forming the negative and positive cohorts enables researchers to easily study the datasets from different angles without the need to prepare a special file for each scenario. Further details on how to utilize the multilevel labeling to construct various scenarios is explained in the “Results and discussion” section.

### Step 3: Data pre-processing

MetaAnalyst provides a variety of pre-processing procedures before downstream statistical analysis. These pre-treatment procedures can be categorized into: (i) filtering, (2) centering, and (3) normalization operations. Filtering aims at removing the variables that are not present in the majority of samples. Removing such under-represented (i.e., absent) variables simplifies and accelerates the downstream analysis. Centering operations convert the abundances to be around zero or median instead of the mean of the microbe abundance levels [45]. Normalization seeks converting the samples to be comparable by removing the systematic variability due to differences in sequence depth. In total, users are provided with one filtering (i.e., removing inactive variables), two centering (i.e., median and zero), and five normalization (i.e., total counts, median, upper quartile, reversed cumulative sum scaling (RCSS), z-score) operations to prepare their input data for subsequent analysis. The detailed information of each pre-processing procedure can be found in the software manual.

### Step 4: Statistical analysis

MetaAnalyst supports two kinds of analysis: (1) biomarker detection, and (2) phenotype classification. For biomarker detection, the MetaAnalyst packs 28 metagenomic biomarker discovery algorithms, namely, Shotgun-FunctionalizeR [19], Boruta [15], edgeR [23], DESeq2 [24], ENNB [16], MetagenomeSeq [17], MicrobiomeDDA [18], MetaStats [20], Raida [21], LEfSe [9], RPCA [10], RegLRSD [11] , RSPCA [46], Lasso [47], Relief [48], ReliefF [49], and the following hypothesis tests: Wilcoxon Rank Sum Test [50], t-Test [51], log t-Test [51], square t-Test [51], Welch’s Test [52], Chi-square Test [53], which are implemented using “stats” package R [30], Kolmogorov Smirnov Test [54], Levene Absolute Test [55], Levene Quadratic Test [55], Brown Forsythe Test [56], BSS/WSS (Between Sum of Squares over Within Sum of Squares) [57], and Pearson Correlation [58], which are implemented using MATLAB. Detailed description of these methods are provided in the User Manual. The biomarker detection phase assigns each variable (i.e., microbe) a score that determines its significance. Then, the top scored variables, according to a predefined number, will be declared as potential markers.

For phenotype classification, the MetaAnalyst package included RF, kNN, four variates of SVM (linear, polynomial, gaussian and radial basis function (RBF)), and two variates of the NCC (namely NCC-1 and NCC-2) classifiers. The difference between NCC-1 and NCC-2 is that the former utilizes the $l_1$ norm to measure the distance, while the second uses the Euclidean distance. These classifiers can be used for (i) building phenotype classification models, and (ii) evaluating the discrimination power of the detected markers. To achieve this, the data corresponding to the identified markers are extracted and used to train and test the classifier using k-fold cross validation.

To provide the user with a comprehensive analysis capability, the MetaAnalyst package enables the user to select multiple biomarker detection algorithms to evaluate different numbers of potential markers at once. Besides, the MetaAnalyst package provides the user with the capability of saving the current simulation settings to be used in future analyses. Also, it enables the user to load the previously saved configuration. This feature helps researchers to generate reusable workflows to compare several algorithms under the same settings and conduct the same analysis over multiple datasets.

Further details about the packed algorithms and the classification measures are provided in the software manual.

### Step 5: Results and plots

MetaAnalyst software provides several publication-quality interactive plots, as listed below, to present the obtained results:

- **Detected biomarkers:** for each BD algorithm and for each number of top features (i.e., biomarkers), the MetaAnalyst presents the identified markers and their scores as a horizontal bar graph. The blue and red bars represent the markers that are enriched in negative and positive class, respectively.
- **Consensus performance** Consensus performance aims at presenting the agreement among different biomarker detection algorithms as an upset plot. This plot shows the overlap between the suggested markers by the BD algorithms included in the analysis.
- **Clustering performance:** Based on the idea that reliable markers are supposed to enlarge the difference between samples belonging to different groups, the two-way unsupervised hierarchical clustering can be utilized to visualize the discrimination power of the biomarker detection algorithm [39]. In particular, the data corresponding to the detected markers are employed to perform hierarchical clustering of samples and selected microbes. This generates a clustering diagram (visualized as a heatmap and two dendrograms, and hence the name two-way clustering), where the rows and columns of the heatmap represent the microbes and samples, respectively. Under such a setting, a reliable biomarker detection algorithm is expected to generate heatmaps with clear separation between the positive and negative cohorts. It is worth to mention that the average linkage and Euclidean distance have been used to generate the dendogram plots. For each BD algorithm and for each number of top features, the MetaAnalyst shows the two-way clustering over the significantly identified differential markers as a heatmap and dendrogram.
- **Classification performance:** To evaluate the classification performance, MetaAnalyst computes the overall classification accuracy (ACC), balanced accuracy (BACC), sensitivity (SEN), specificity (SPC), miss classification rate (MCR), receiver operation curve (ROC), and area under the curve (AUC). These metrics capture various aspects of the classification performance. For example, the accuracy (the ratio of the correctly detected samples in both classes) is biased toward the class with dominant samples. Therefore, for extremely skewed datasets, the accuracy may be misleading, and hence class-specific measures (e.g., sensitivity and specificity) or BACC may be more reliable to account for bias. MetaAnalyst displays the seven classification performance metrics (i.e., ACC, BACC, SPC, SEN, ROC, AUC, MCR) for all the included algorithms in the analysis.

To enhance the user’s experience, the MetaAnalyst software provides the user with the flexibility to control various settings of the generated plots such as the size of the plots, description of the axis (i.e., x-label and y-label), the title of the figure, the fontsize, etc. After finalizing the figure formatting, the user can save the plots in thirteen different formats: jpg, png, tif, pdf, fig, eps, bmp, emf, pcx, pbm, pgm, ppm, svg. In addition to the generated plots, the user can export all the results as excel sheets.

**Fig. 2:**
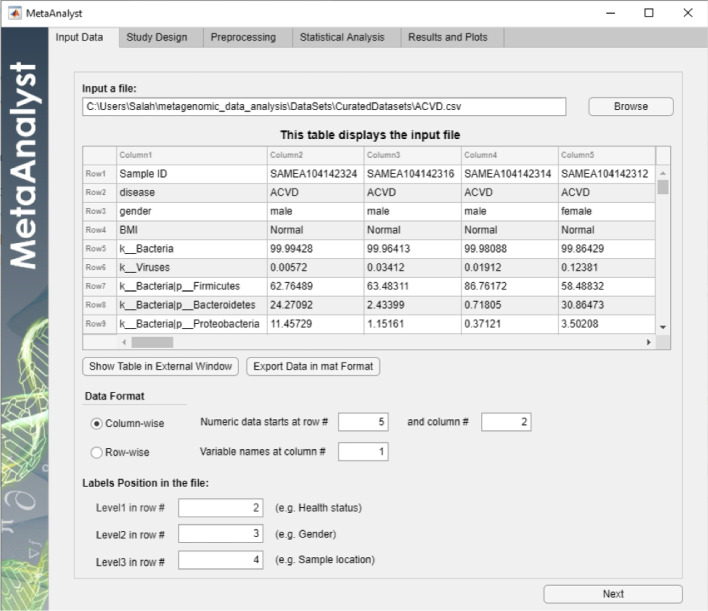
Input tab

**Fig. 3:**
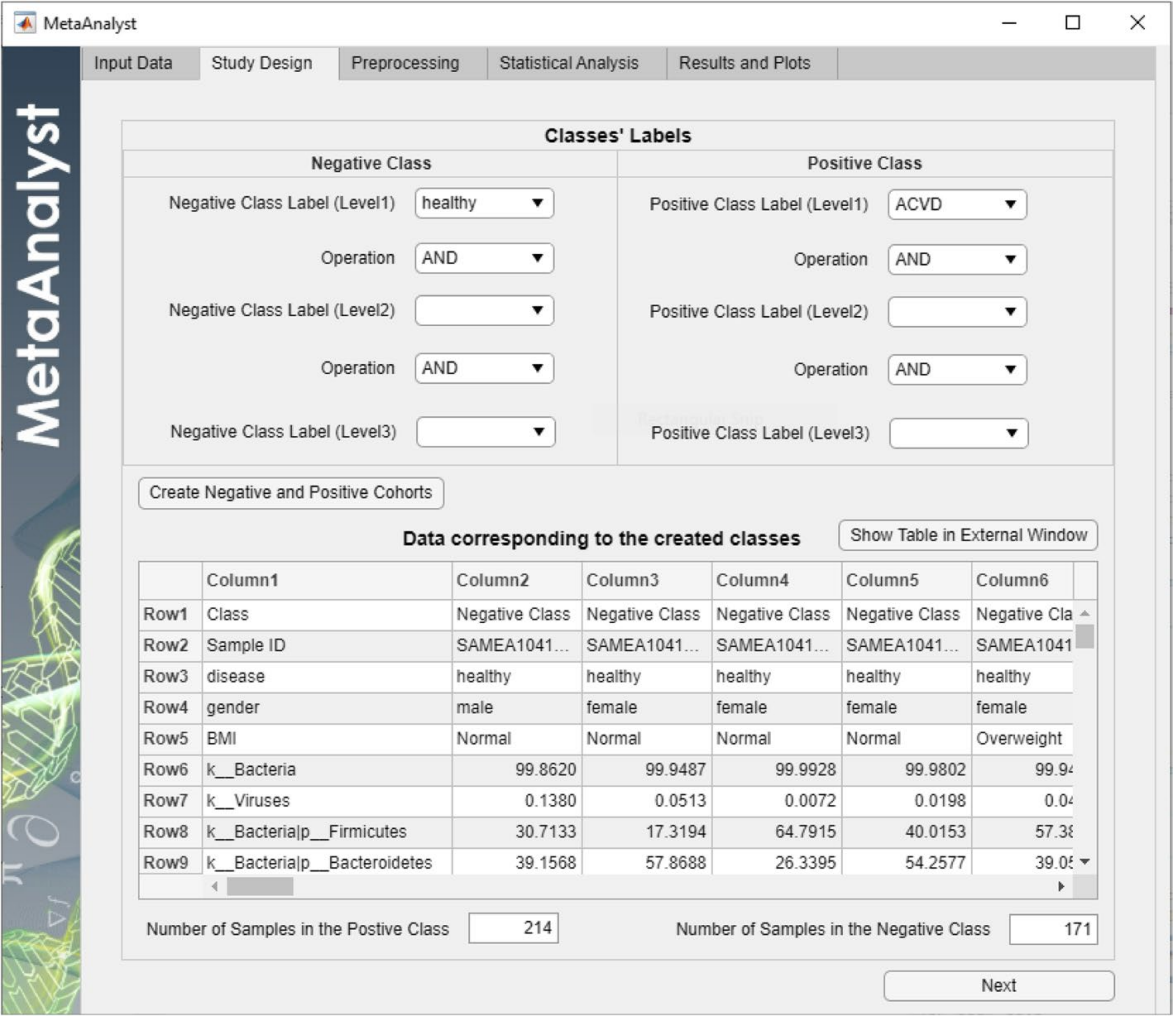
Study design tab for Scenario 1

**Fig. 4:**
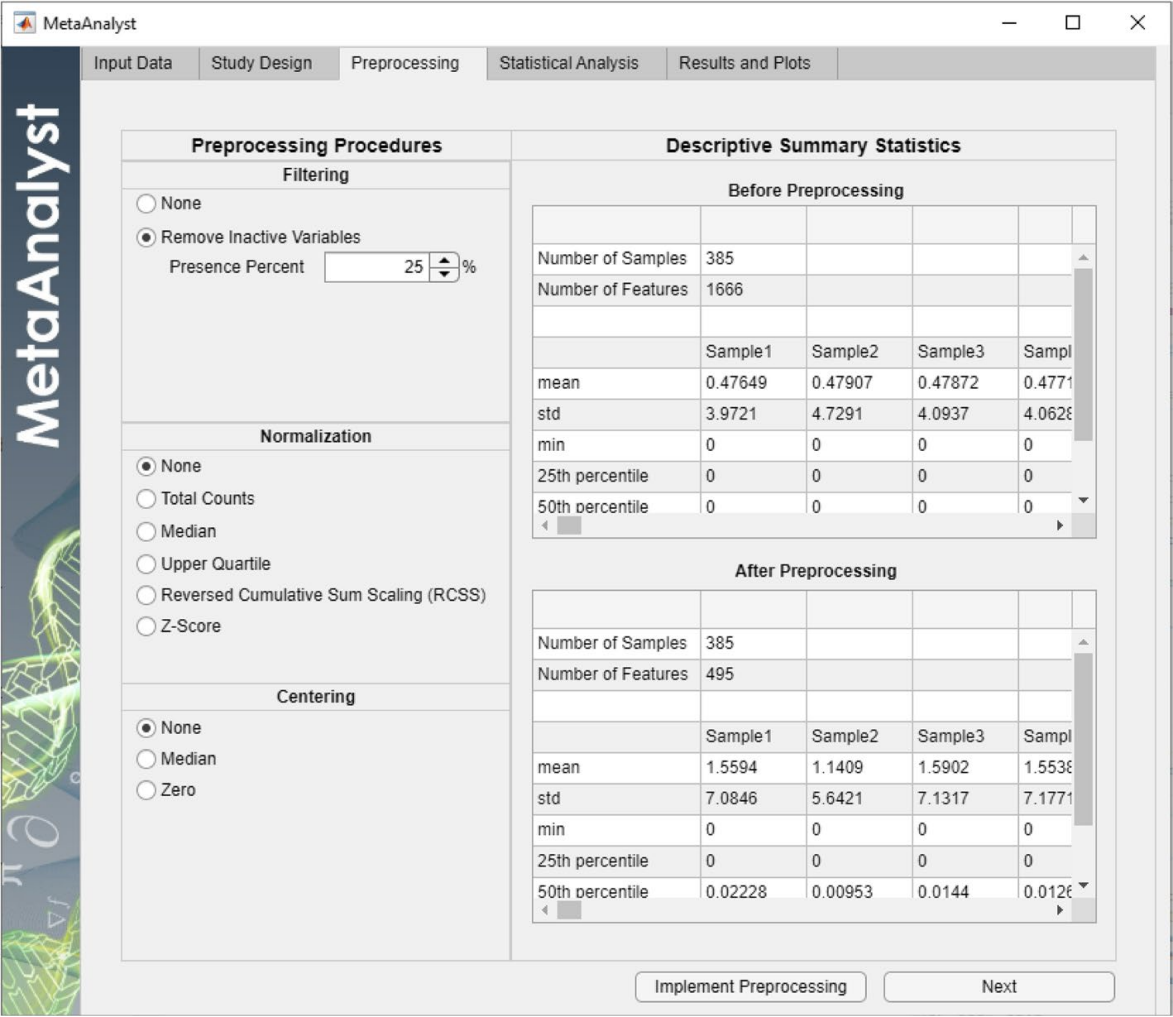
Preprocessing tab for Scenario 1

**Fig. 5:**
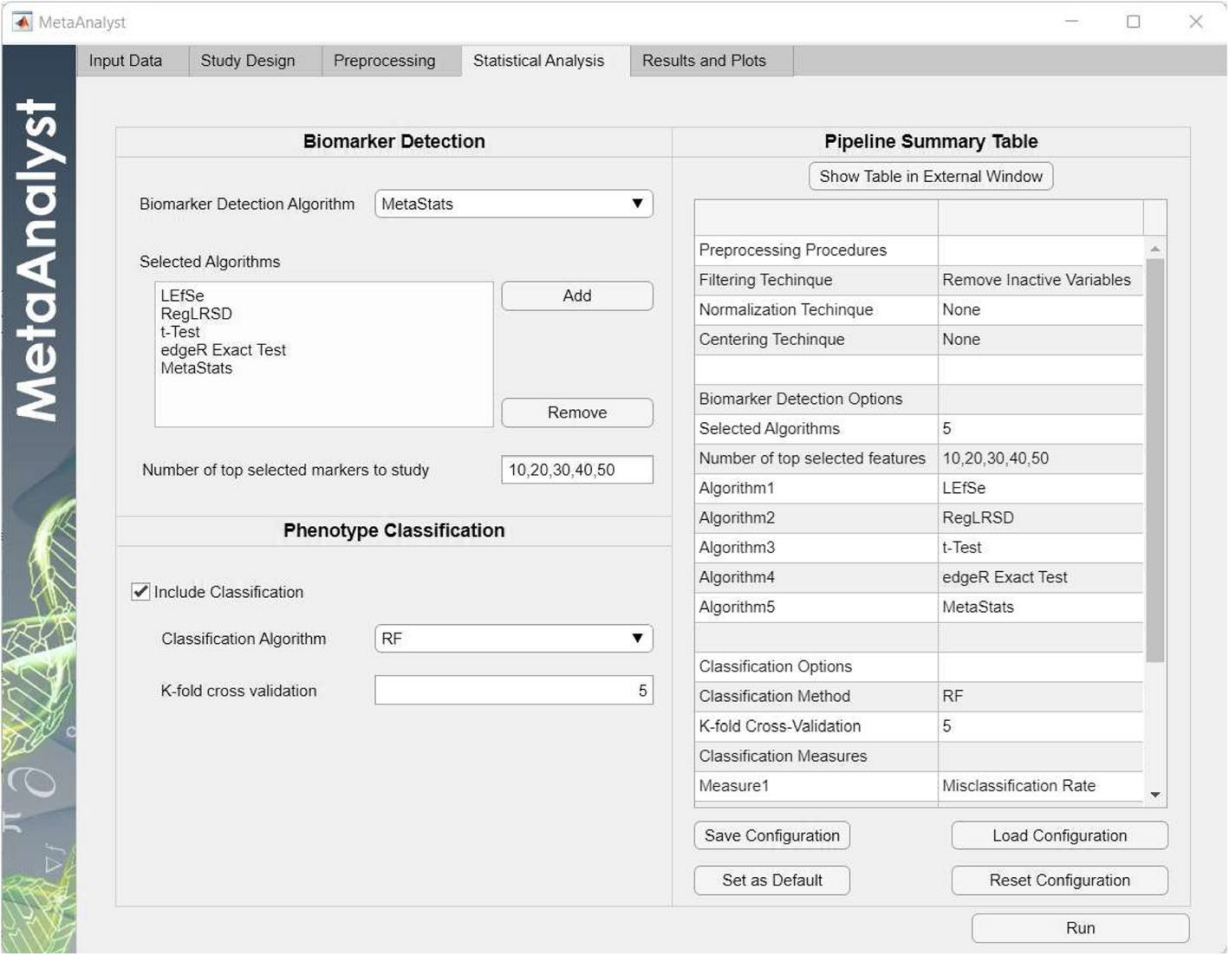
Statistical analysis tab for Scenario 1

**Fig. 6:**
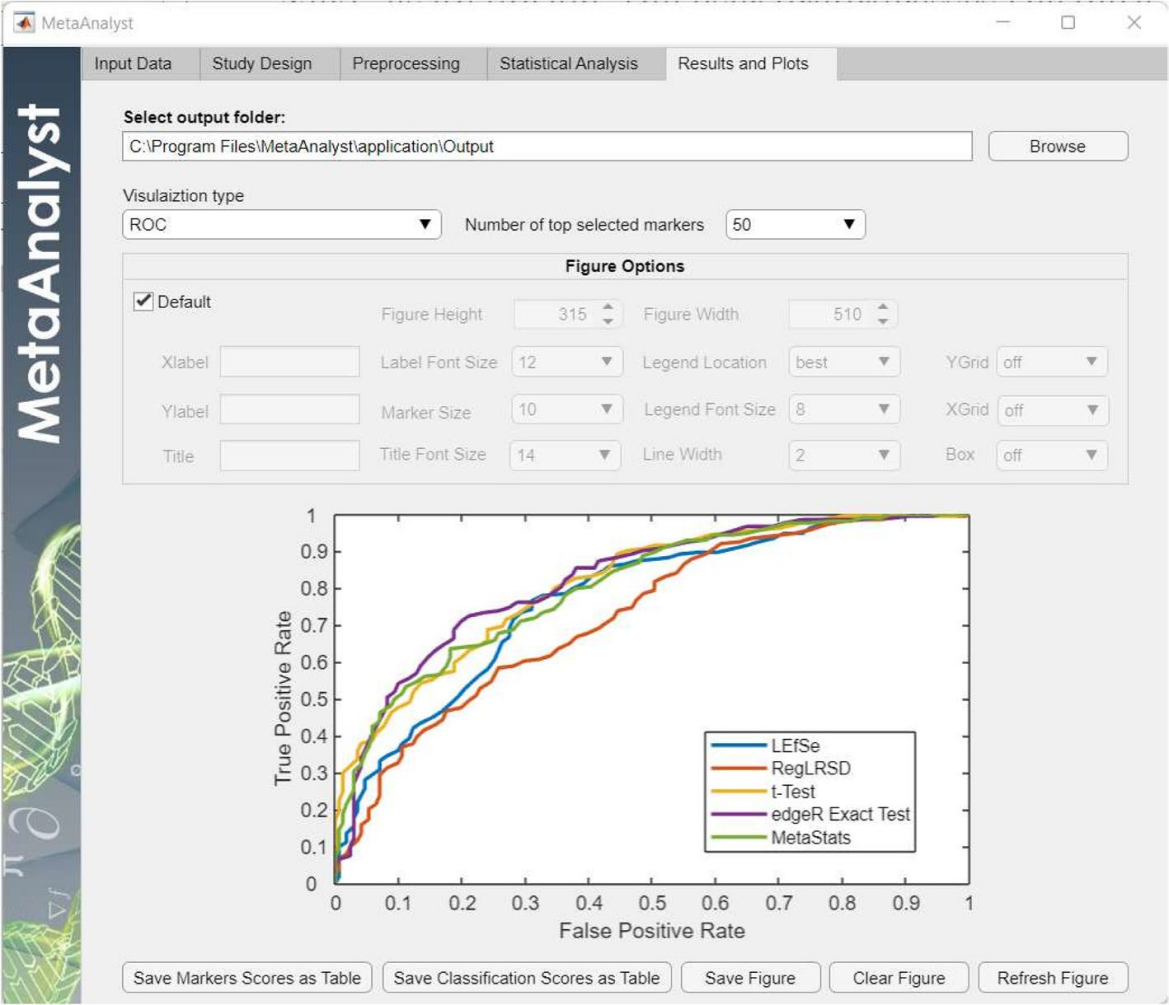
Results and plots tab for Scenario 1

## Results and Discussion

This section demonstrates the flexibility and ease-of-use of MetaAnalyst by analyzing a metagenomic dataset related to acute cardiovascular disease (ACVD) under various scenarios/conditions. This dataset studies the relationship between human gut microbiota and ACVD [59]. The dataset is composed of metagenomic stool samples from 218 ACVD patients and 187 healthy subjects. A snapshot of the loaded dataset as displayed by MetaAnalyst is shown in Fig. 2.

As can be seen in Fig. 2, each sample is annotated with three levels of labels: level 1: disease status (in row 2), level 2: gender (in row 2), and level 3: BMI status (in row 3). It is worth to mention that it is not required to fill the information about the location of all levels of labels. It is required only to locate the levels that the user is interested in his/her study. For example, assume that the researcher is interested only on the effect of disease and BMI status, as discussed in the following two subsections, then it is necessary to specify only the rows that store the labels of disease and BMI status. Besides, the loaded data as shown in Fig. 2 is in column-wise format (each column represents one sample), the numeric data starts at row 5 and column 2, and the variable names resides at column 1.

To demonstrate the capability of MetaAnalyst to study the dataset from different perspectives, we consider four scenarios as summarized in Table 2.

**Table 2:** Four possible scenarios to study the ACVD dataset

	Positive Class	Negative Class
Scenario 1	Diseased	Healthy
Scenario 2	Diseased & Normal	Healthy & Normal
Scenario 3	Diseased & Normal & Female	Healthy & Normal & Female
Scenario 4	Diseased & Normal & Male	Healthy & Normal & Male

**Fig. 7:**
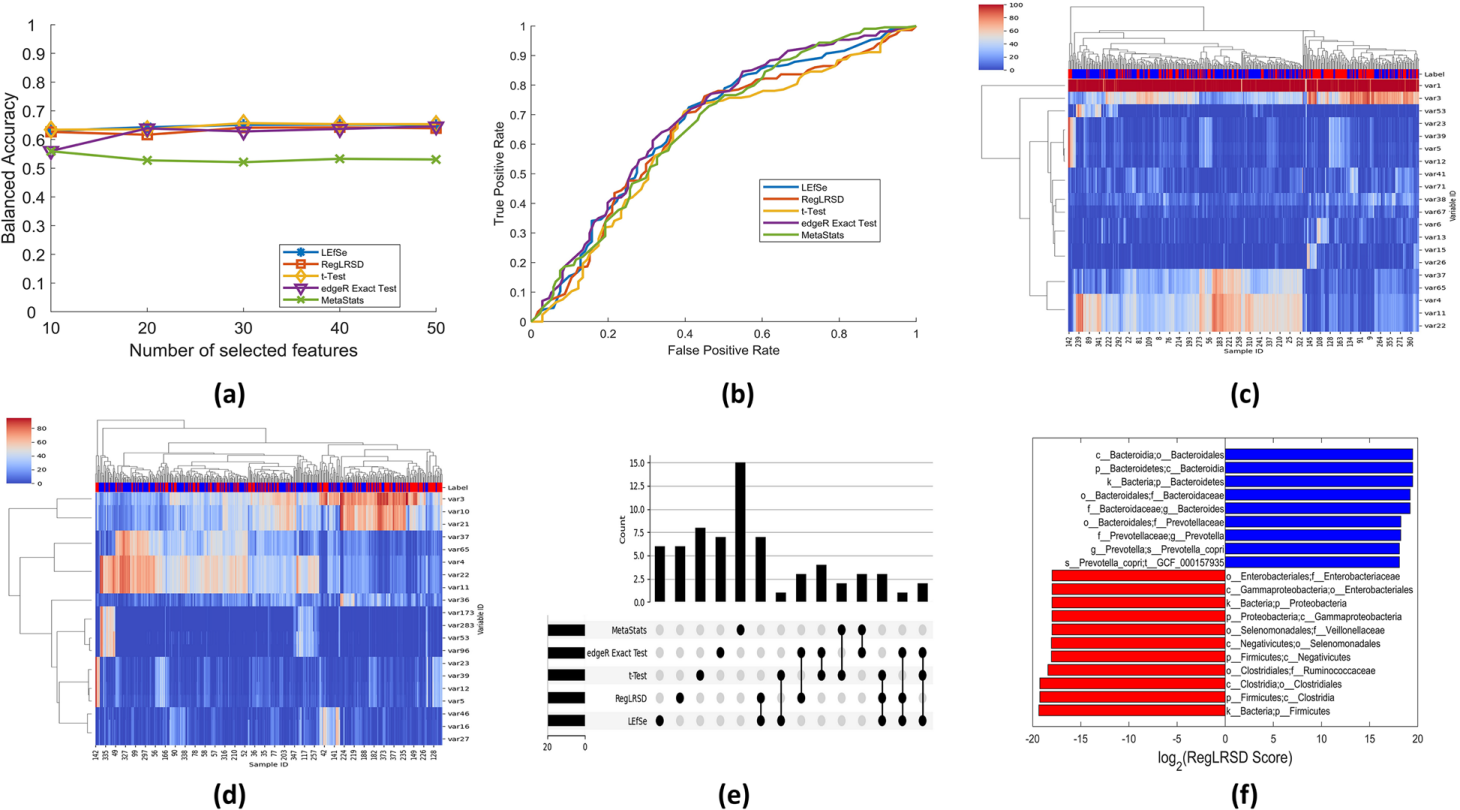
Sample of the obtained results over Scenario 1. (a) &(b) Achieved BACC and ROC for the selected biomarker detection algorithms: LEfSe, RegLRSD, t-Test, Exact-Test (edgeR), and MetaStats, respectively. (c) and (d) The unsupervised clustering performance of the top 20 markers as suggested by edgeR and MetaStats algorithms, respectively. (e) The number of overlapped potential markers among the five BD algorithms. (f) Suggested markers by the LEfSe algorithm

**Fig. 8:**
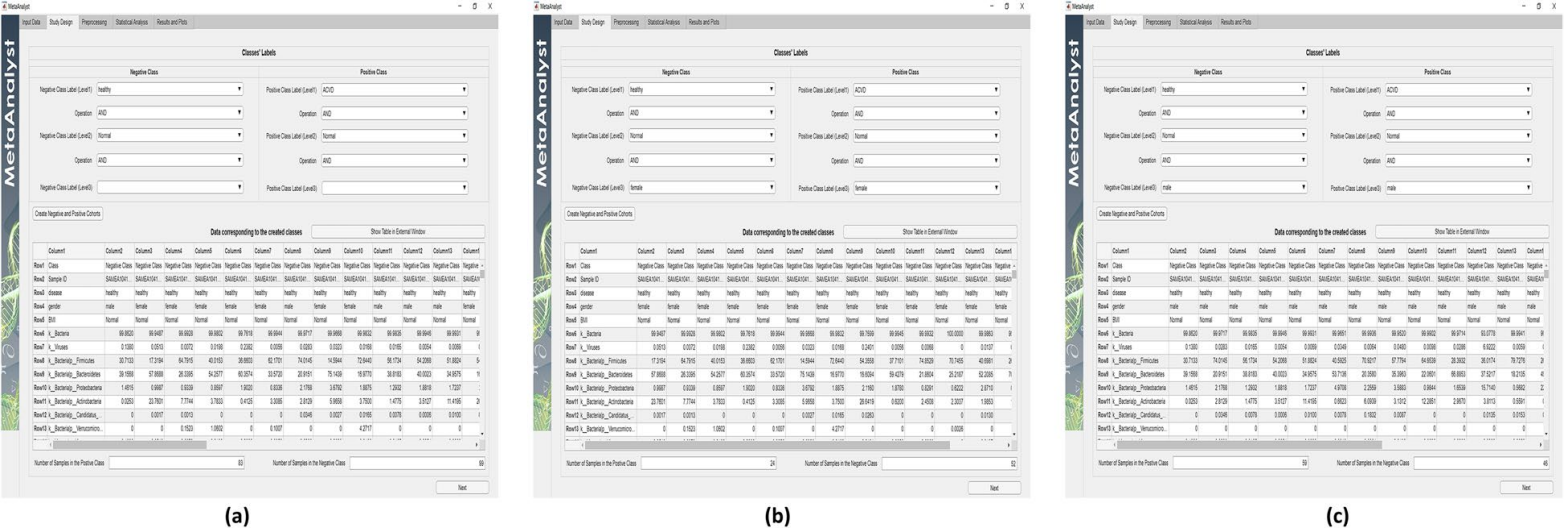
Construct scenarios 2, 3, and 4 in Table 2 using the multi-level labeling property supported in the “Study Design” tab

**Fig. 9:**
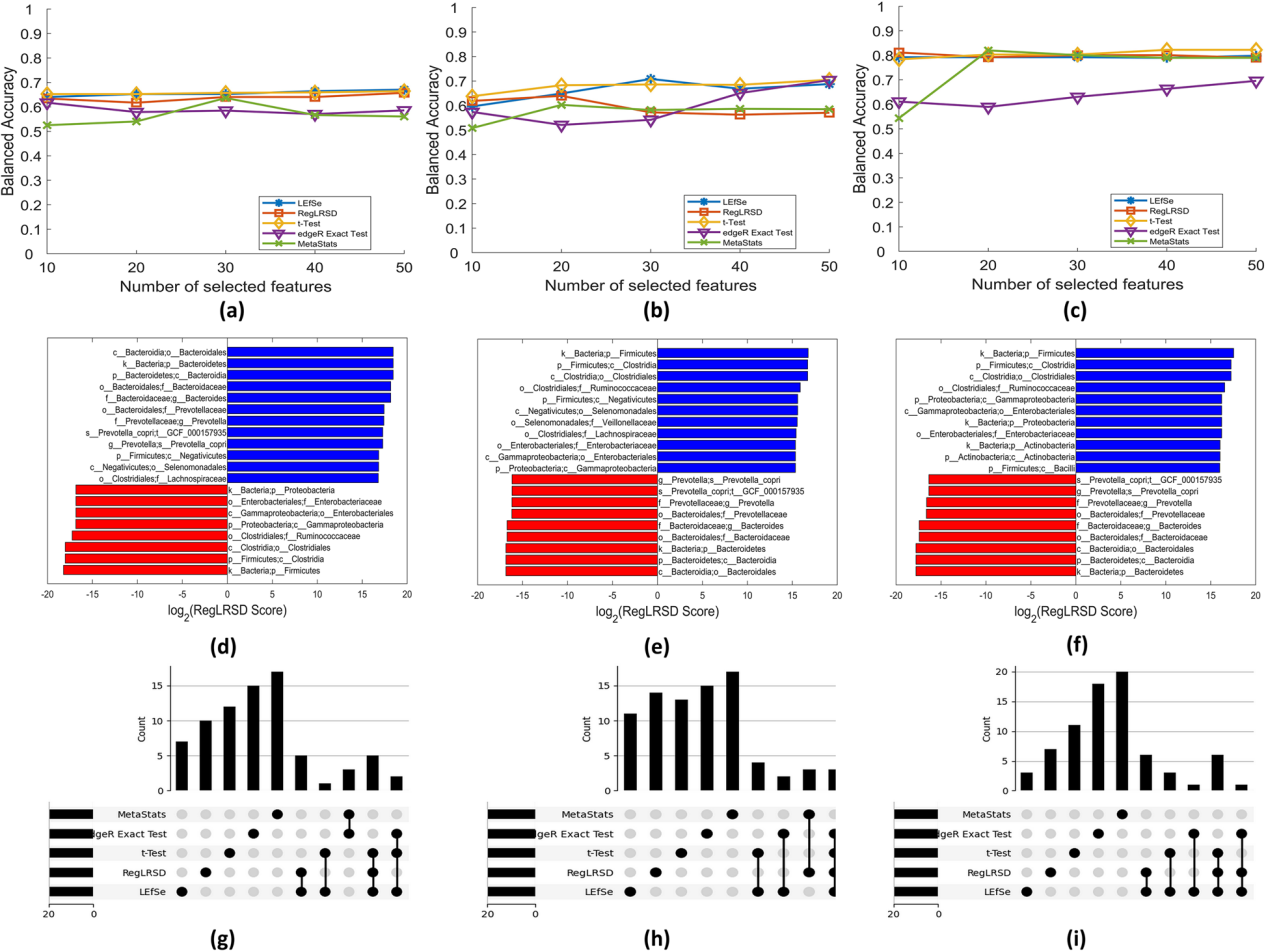
Sample of the obtained results, using LEfSe, RegLRSD, t-Test, edgeR, and MetaStats algorithms, that studies the impact of obesity and gender over the ACVD dataset (i.e., scenarios 2, 3, and 4 in Table 2). The results in the first, second and third column correspond to scenario 2, 3, and 4, respectively. The first row displays the achieved BACC performance over the three scenarios. The second row shows the suggested 20 markers by the RegLRSD algorithm. The third row presents the overlapping between the suggested 20 markers by the five BD algorithms

### Healthy versus diseased

Initially, let us consider the first scenario that aims at finding potential markers that discriminate between healthy and diseased subjects irrespective of their gender and obesity status (BMI value). Therefore, in the “Study Design” tab, the negative and positive classes are set, using only level 1, to include “healthy” and “ACVD” subjects, respectively, and left the other two levels (i.e., level 2: gender and level 3: BMI status) empty as shown in Fig. 3.

Next, as an option, the user preprocesses the data before the downstream analysis using the options in the “Preprocessing” tab shown in Fig. 4. In this study, we chose to only remove all inactive variables that are not present in at least 25% of the samples of either class. This reduces the number of variables from 1666 to only 495. Furthermore, the “Preprocessing” tab displays two tables presenting summary statistics about the number of samples, number of features, mean, standard deviation, minimum, $25^{th}$ percentile, $50^{th}$ percentile, $75^{th}$ percentile and the maximum value for each sample.

Upon preparing the data, the user can design the analysis work-flow by selecting the biomarker detection algorithms from the drop-down list as shown in Fig. 5. As mentioned earlier, the user can select multiple detection algorithms for various number of top features to conduct the analysis over all of them simultaneously. In this study, 5 biomarker detection algorithms (LEfSe, RegLRSD, t-Test, edgeR, and MetaStats) were included in the analysis. Besides, the user can extend the biomarker detection algorithm to include a classifier model. This classifier is used for both (1) evaluating the performance of biomarker detection algorithms and (2) building a phenotype classification model. In this experiment, the NCC-1 classifier was employed. The classification performance is estimated using 5-fold cross-validation. MetaAnalyst shows a pipeline summary describing the designed analysis workflow. To enhance the user experience and the reproducibility of the results, the MetaAnalyst software provides the user with the capability to set the current analysis work-flow as a default configuration. Also, the user can save multiple configurations (i.e., analysis workflows) and load the suitable configuration for future analysis.

The user can select the type of the results to be visualized from the “Visualization type” drop list in the “Results and plots” tab as shown in Fig. 6. A sample of these plots is displayed in Fig. 7. For example, the MetaAnalyst shows the detected markers along with their scores in bar graph plots as shown in Fig. 7-a. The unsupervised clustering performance is presented as a tow-way clustering heatmap as shown in 7-b. The achieved classification performance in terms of the BACC is depicted in Fig. 7-c. In addition to the BACC, the MetaAnalyst generates similar plots to ACC, SPC, SEN, ROC, and AUC. The agreement between biomarker detection algorithms is depicted in Fig. 7-d. The algorithm recommending more overlapped markers is expected to be more accurate.

The complete set of results that re generated by the MetaAnalyst software for scenario 1 is depicted in the Additional file 1. In addition to the generated plots, the user can export the classification performance [see Additional file 2], detected markers by each algorithm [see Additional file 3], and the overlapped set of markers [see Additional file 4] as excel sheets.

### Impact of obesity and gender

To shed insights on the utility of the multi-level labeling feature of MetaAnalyst in enabling the study of the dataset from different perspectives, this section demonstrates how to extend the previous scenario to study the impact of obesity and gender. To illustrate, assume that the researcher is interested in excluding the impact of obesity from the previous study (i.e., Scenario 2 in Table 2). That is, to compare healthy versus diseased samples over only normal subjects (i.e., subjects with BMI values in the range 18.9-25). Constructing this study can be achieved simply by setting the operation between the first and second levels to “AND” and select the label to be “Normal” for both negative and positive cohorts as shown in Fig. 8-a. Furthermore, assume that the researcher is interested in extending this study to investigate whether the microbial patterns differ between female ((i.e., Scenario 3 in Table 2)) and male ((i.e., Scenario 4 in Table 2)) individuals. Again, these two studies can be constructed easily by proper setting of the study design tab as shown in Figs. 8-b and 8-c, respectively.

A sample of the obtained results under scenarios 2, 3 and 4 is depicted in Fig. 9. As it can be observed from Figs. 9-a, 9-b, and 9-c, the achieved BACC performance by the five BD algorithms in male subjects is generally higher than female subjects, especially when using LEfSe, RegLRSD, and t-Test algorithms for biomarker detection. Interestingly, male individuals present higher discrimination power compared to females. This result may indicate that the bacterial composition in males present stronger variation compared to females in response to ACVD. Indeed, this observation needs further investigations to evaluate the gender effect on the interaction between human microbiota and cardiovascular disease. This suggests that potential treatments may need to be gender-specific to account for the gender association with ACVD risk factors. Thus, the multi-level labeling feature of MetaAnalyst allows such observations to be easily visualized and detectable.

## Conclusions

This work proposed MetaAnalyst, a stand-alone software package for metagenomic biomarker detection and phenotype classification. The MetaAnalyst package aims at reducing the programming skills and simplifying the tasks required to analyze metagenomic datasets. The MetaAnalyst package (i) automatically installs and handles all package dependencies-related issues of 28 state-of-the-art biomarker detection algorithms and 4 classification models with several data preprocessing capabilities, (ii) provides a simple graphical user interface that naturally guides the user through the analysis pipeline, (iii) accepts input datasets in several files with flexible data formats, (iv) supports multi-level labeling feature to flexibly cluster the positive and negative cohorts and to study a given dataset under a multitude of scenarios, (v) runs several algorithms simultaneously and evaluates their performance according to three criteria (classification, clustering, and overlapping performance), (vi) reports the results in publishing-quality plots as well as Excel sheets. Due to the similarity between metagenomic data and other omic data and the possibility of applying the packed algorithms in MetaAnalyst to other omic data, we believe that MetaAnalyst will become a popular tool for metagenomics applications and other studies. The executable file for MetaAnalyst along with a detailed user manual are made available at https://github.com/mshawaqfeh/MetaAnalyst.

## Supplementary Information

**Additional file 1.** The complete set of plots that are generated by the MetaAnalyst software for scenario 1.

**Additional file 2.** An Excel sheet that is generated by the MetaAnalyst software that contains the obtained classification performance (in terms of MCR, SEN, SPC, ACC, BACC, ROC and AUC) by the five algorithms included in the study (i.e., LEFSe, RegLRSD, t-Test, EdgeR Exact Test, and MetaStats) over Scenario 1 for various number of top features (i.e., 10, 20, 30, 40, and 50).

**Additional file 3.** An Excel sheet that is generated by the MetaAnalyst software that contains the top 20 detected metagenomic markers by the five algorithms included in the study (i.e., LEFSe, RegLRSD, t-Test, EdgeR Exact Test, and MetaStats) over Scenario 1.

**Additional file 4.** An Excel sheet that is generated by the MetaAnalyst software that contains the overlapped set of metagenomic markers by the five algorithms included in the study (i.e., LEFSe, RegLRSD, t-Test, EdgeR Exact Test, and MetaStats) over Scenario 1.

## Data Availability

$\bullet$ Project name: MetaAnalyst $\bullet$ Project home page: https://github.com/mshawaqfeh/MetaAnalyst $\bullet$ Operating system(s): Microsoft Windows $\bullet$ Programming language: Matlab, R and Python $\bullet$ Other requirements: No requirements $\bullet$ License: MetaAnalyst is made readily available freely to any scientist wishing to use it for non-commercial purposes, without any restriction. $\bullet$ Any restrictions to use by non-academics: license needed

## Abbreviations

ACC: Accuracy
ACVD: Acute Cardiovascular Disease
AUC: Area Under the Curve
BACC: Balanced Accuracy
BD: Biomarker Detection
GUI: Graphical User Interface
IBD: Inflammatory Bowel Disease
kNN: k-Nearest Neighbor
MCR: Miss Classification Rate
OTU: Operational Taxonomic Unit
RCSS: Reversed Cumulative Sum Scaling
RF: Random Forest
ROC: Receiver Operation Curve
SEN: Sensitivity
SPC: Specificity
SVM: Support Vector Machine
