# Supplementary material for: MetaAnalyst: a user-friendly tool for metagenomic biomarker detection and phenotype classification
Source: BMC Med Res Methodol. 2022 Dec 28;22:336. doi: 10.1186/s12874-022-01812-5 (PMC9795700; doi:10.1186/s12874-022-01812-5)
Supplement: Supplementary file 1 — Additional file 1. The complete set of plots that are generated by the MetaAnalyst software for scenario 1. [file 12874_2022_1812_MOESM1_ESM.pdf]

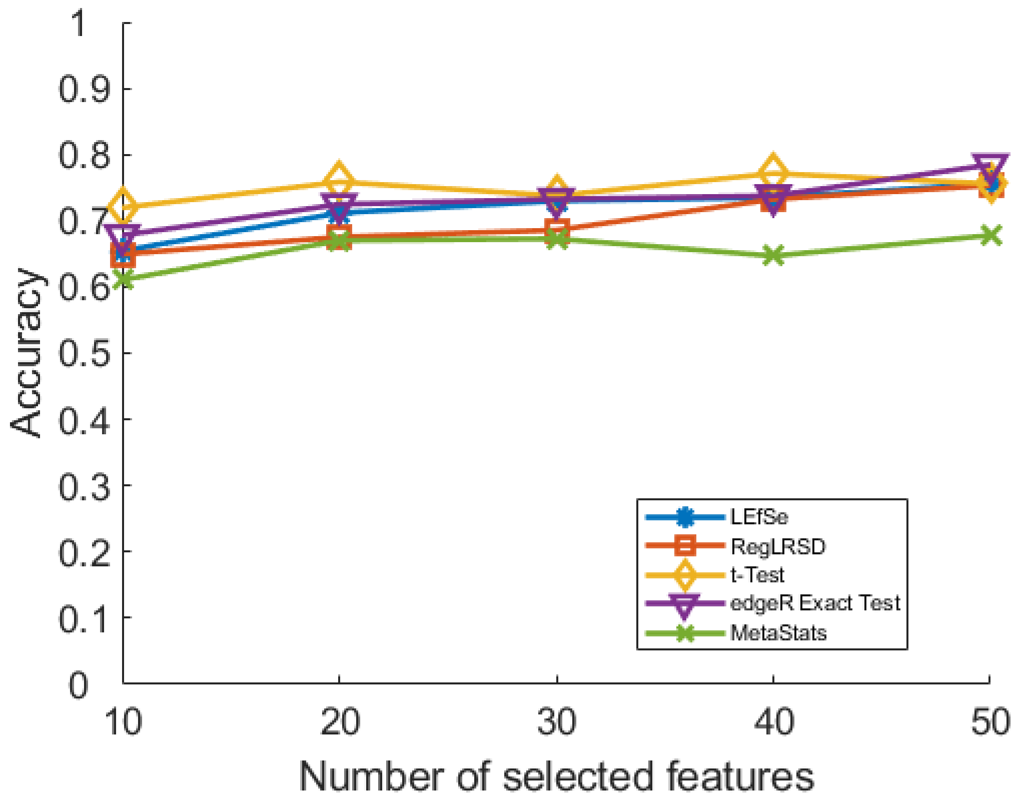

Fig. S1: Achieved ACC for the selected biomarker detection algorithms: LEfSe, RegLRSD, t-Test, Exact-Test (edgeR), and MetaStats, respectively.

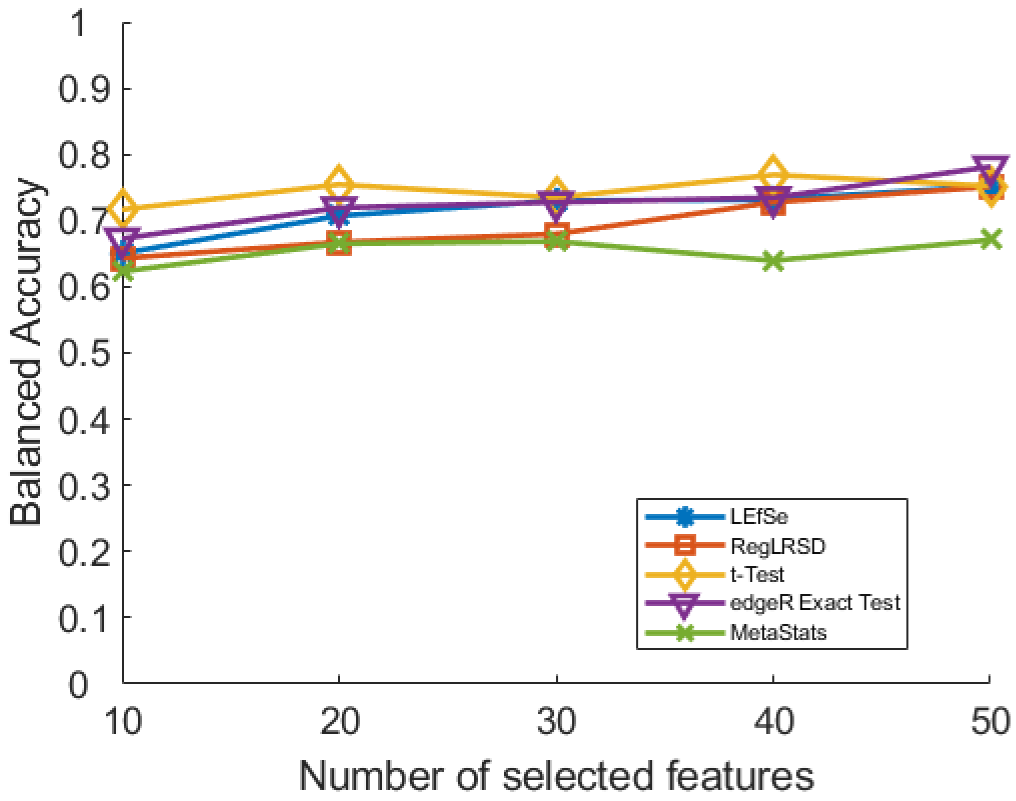

Fig. S2: Achieved BACC for the selected biomarker detection algorithms: LEfSe, RegLRSD, t-Test, Exact-Test (edgeR), and MetaStats, respectively.

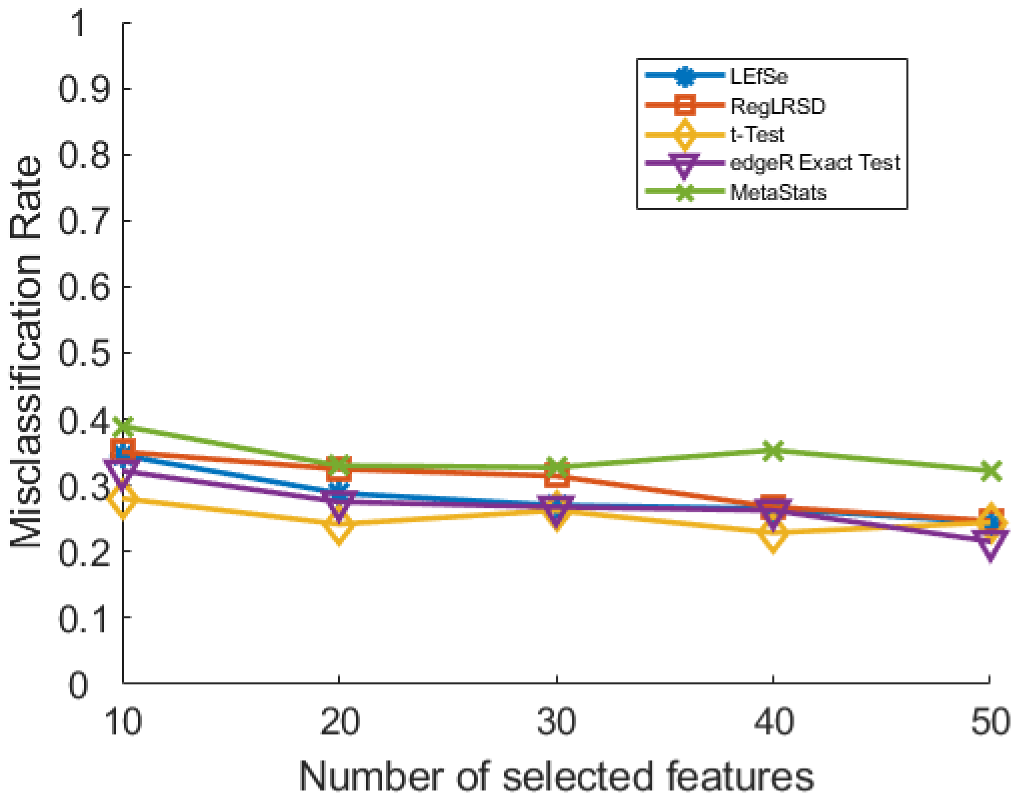

Fig. S3: Achieved MCR for the selected biomarker detection algorithms: LEfSe, RegLRSD, t-Test, Exact-Test (edgeR), and MetaStats, respectively.

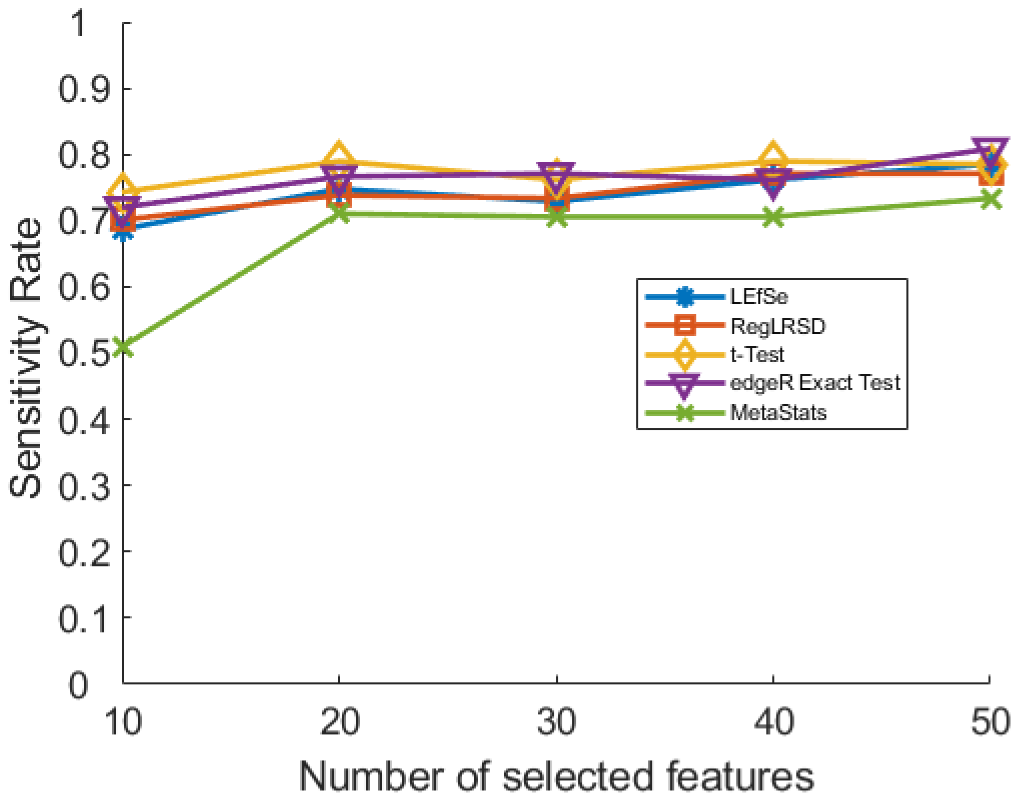

Fig. S4: Achieved sensitivity for the selected biomarker detection algorithms: LEfSe, RegLRSD, t-Test, Exact-Test (edgeR), and MetaStats, respectively.

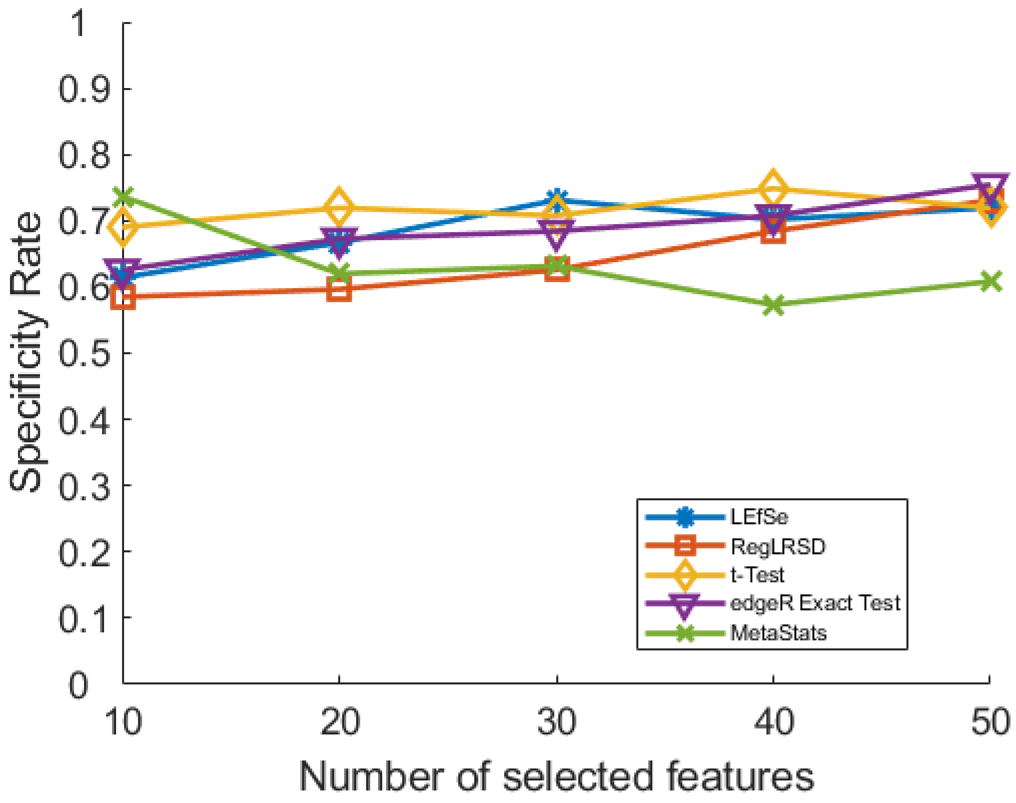

Fig. S5: Achieved specificity for the selected biomarker detection algorithms: LEfSe, RegLRSD, t-Test, Exact-Test (edgeR), and MetaStats, respectively.

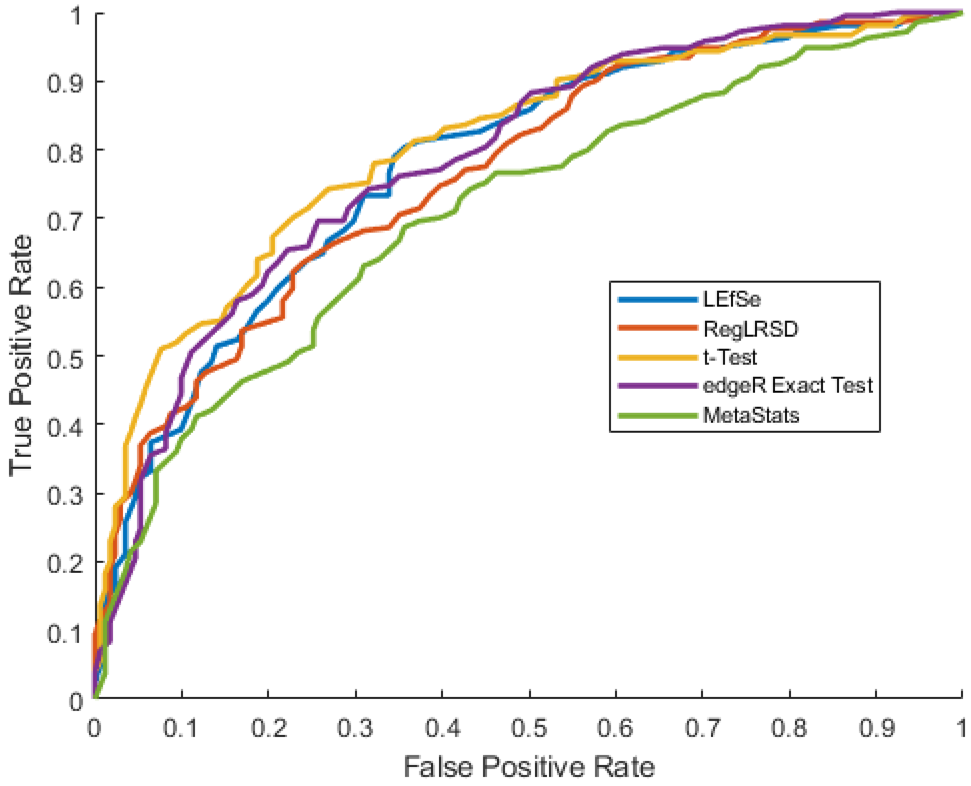

Fig. S6: Achieved ROC for the selected biomarker detection algorithms: LEfSe, RegLRSD, t-Test, Exact-Test (edgeR), and MetaStats, respectively.

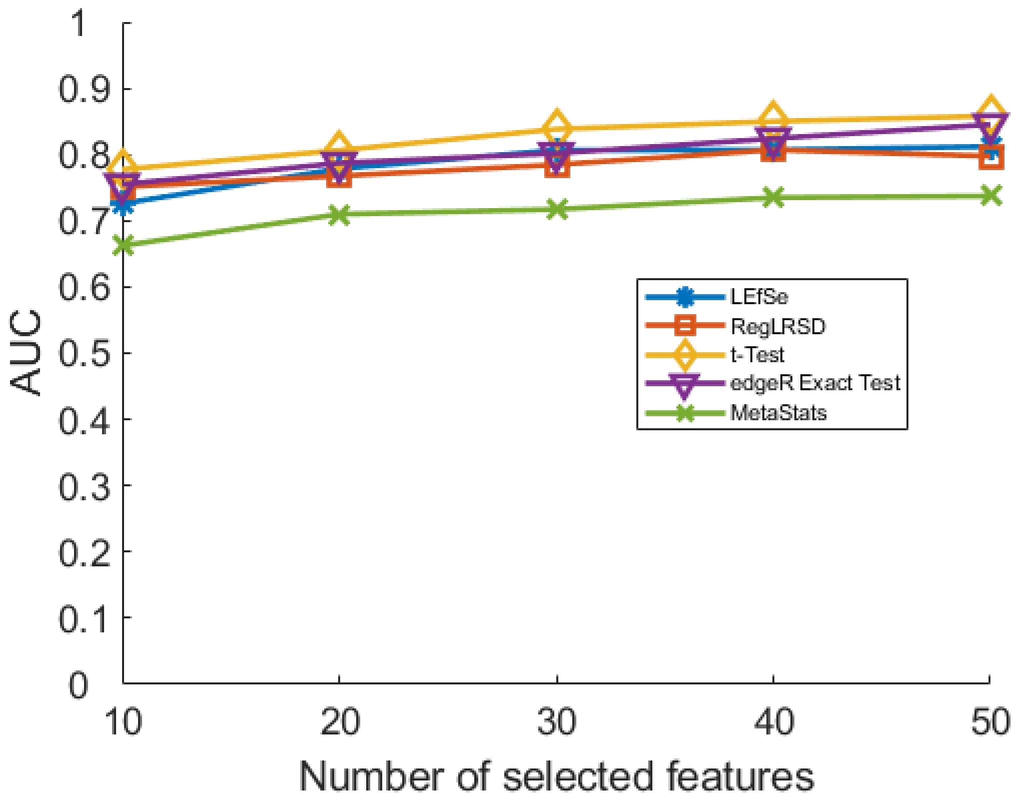

Fig. S7: Achieved AUC for the selected biomarker detection algorithms: LEfSe, RegLRSD, t-Test, Exact-Test (edgeR), and MetaStats, respectively.

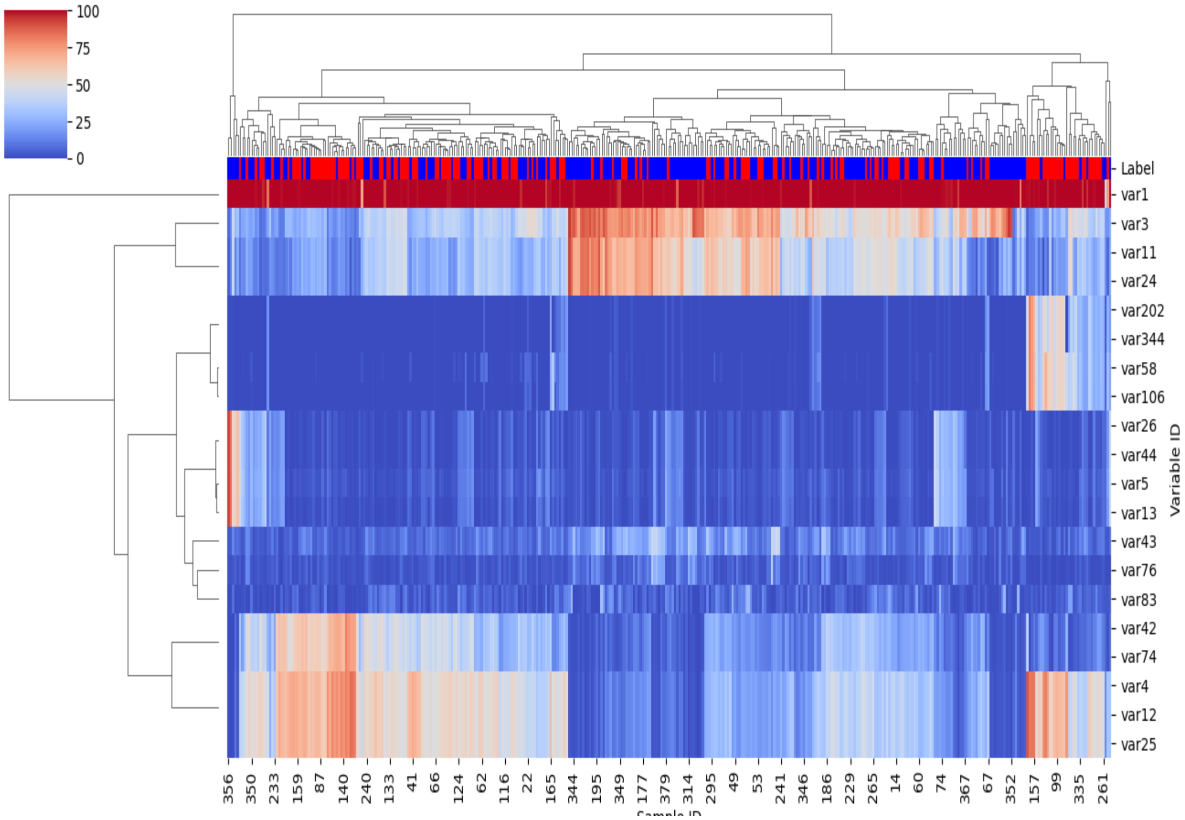

Fig. S8: The unsupervised clustering performance of the top 20 markers as suggested by LEfSe algorithm.

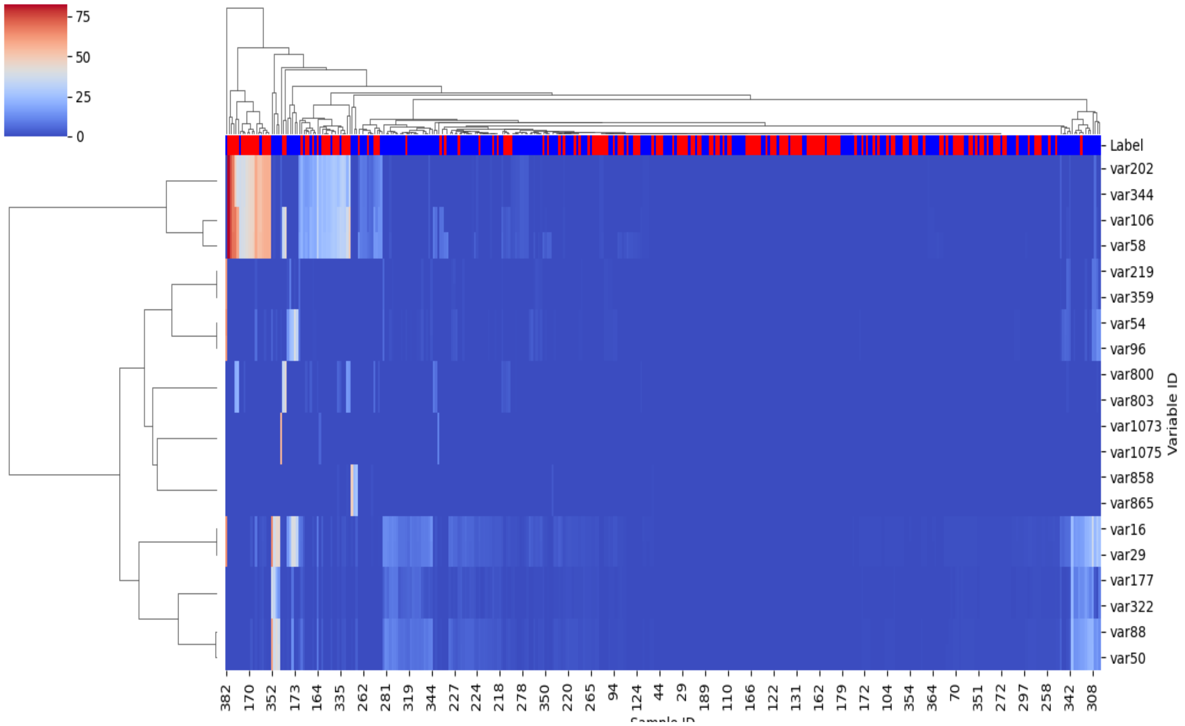

Fig. S9: The unsupervised clustering performance of the top 20 markers as suggested by edgeR (exact test).

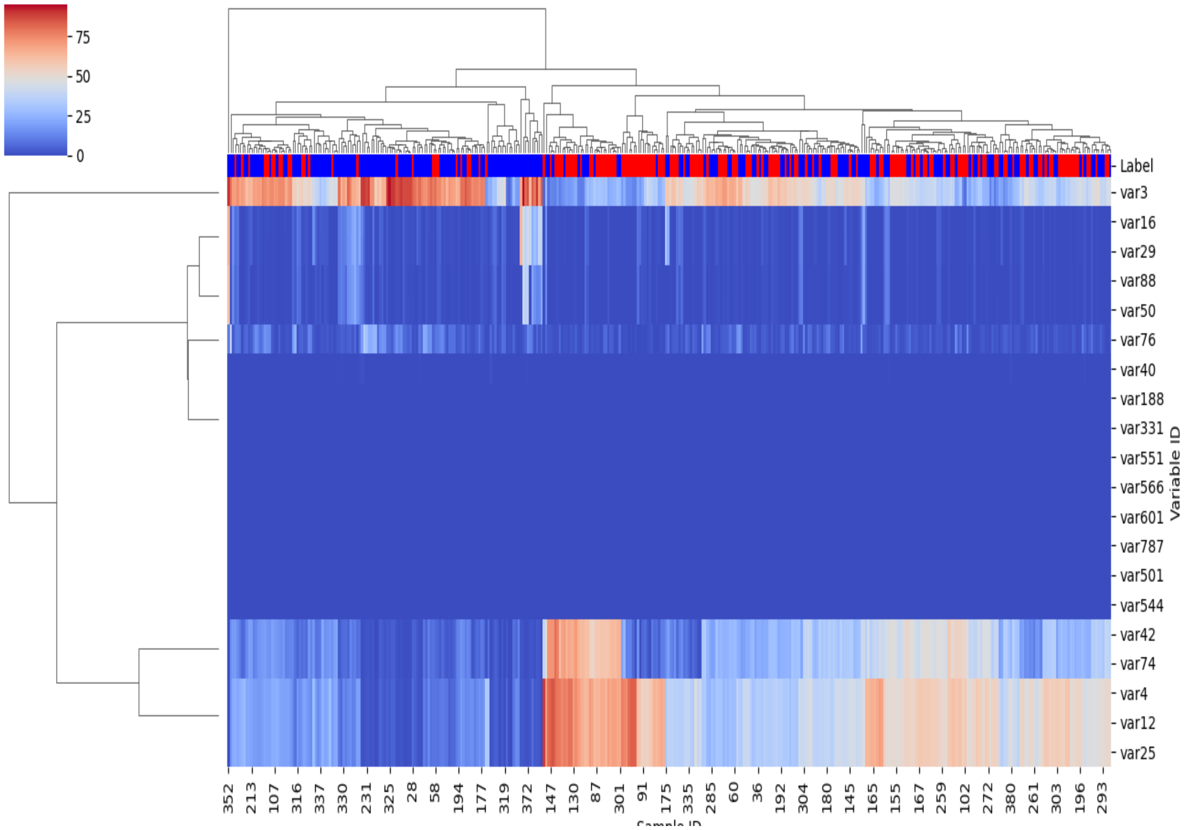

Fig. S10: The unsupervised clustering performance of the top 20 markers as suggested by t-test.

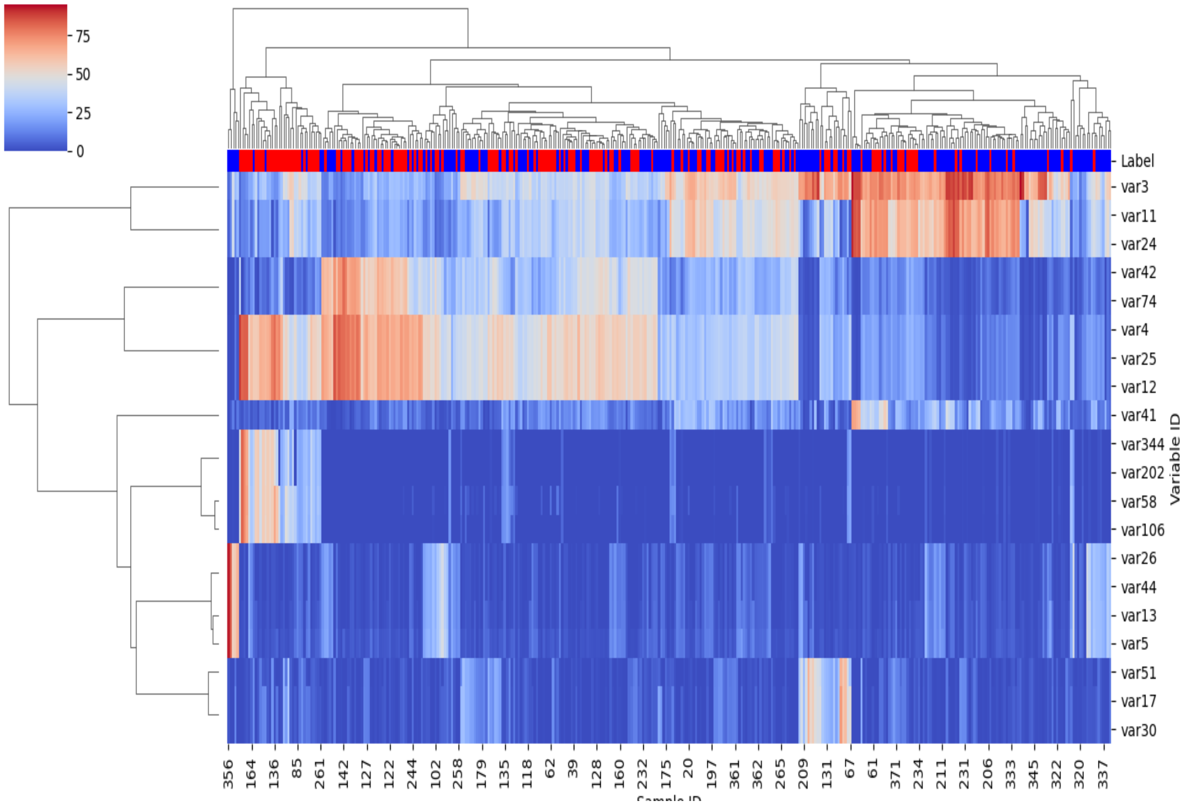

Fig. S11: The unsupervised clustering performance of the top 20 markers as suggested by RegLRSD algorithm.

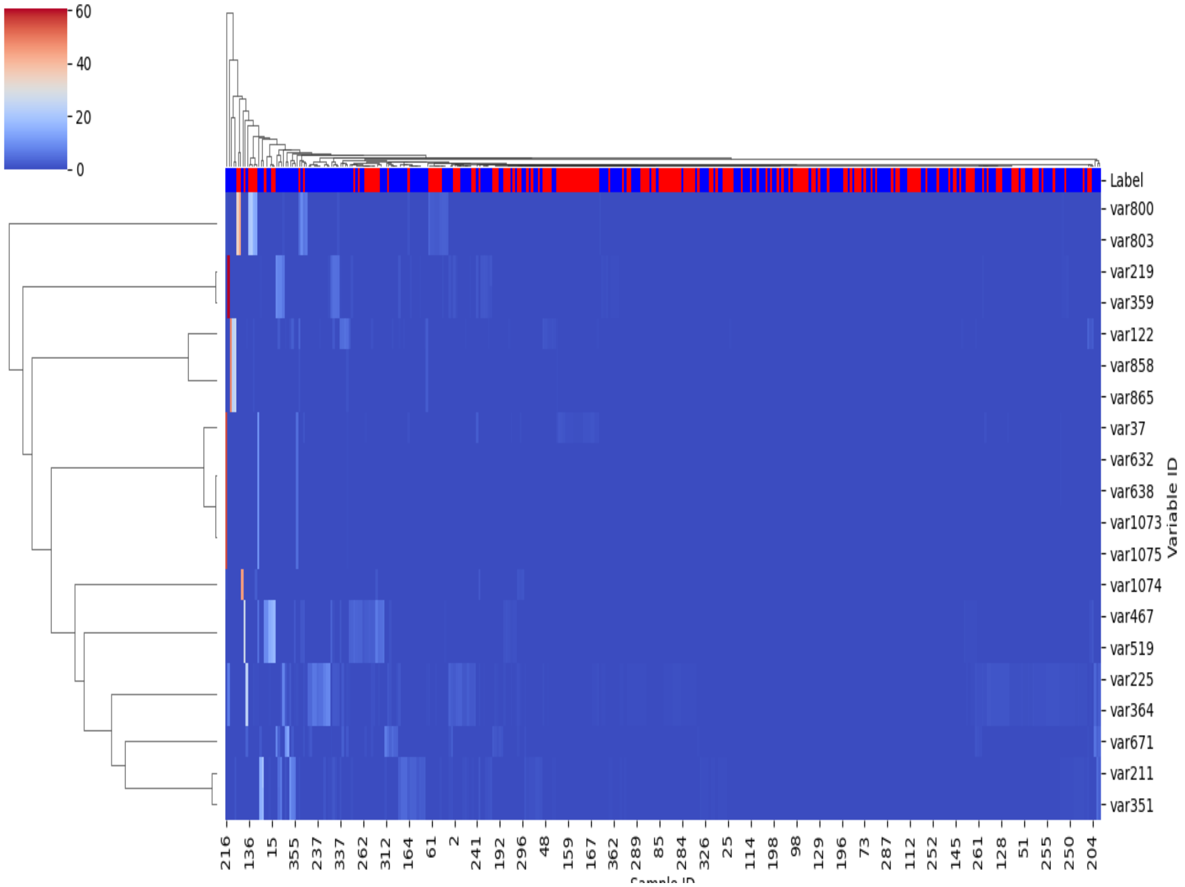

Fig. S12: The unsupervised clustering performance of the top 20 markers as suggested by MetaStats algorithm.

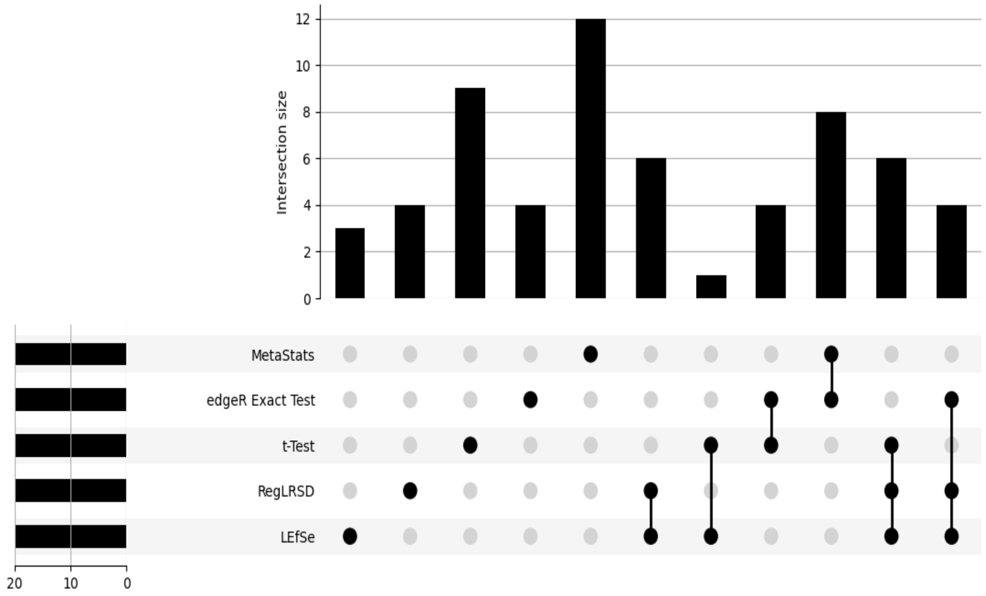

Fig. S13: The number of overlapped potential markers among the five BD algorithms..
